# Editorial: Receptors in cardiovascular diseases: Mechanisms, diagnosis, and treatment

**DOI:** 10.3389/fcvm.2023.1177727

**Published:** 2023-03-24

**Authors:** Shutong Shen, Francisco J. Rios, Phung N. Thai

**Affiliations:** ^1^ Department of Cardiology, Research Unit of Cardiovascular Techniques and Devices, Chinese Academy of Medical Sciences, Zhongshan Hospital, Fudan University, Shanghai, China; ^2^ Shanghai Institute of Cardiovascular Diseases, Shanghai, China; ^3^ Research Institute of the McGill University Health Centre (RI-MUHC), Montreal, QC, Canada; ^4^ Division of Cardiology, Department of Internal Medicine, University of California, Davis, CA, United States

**Keywords:** cardiovascular disease, receptors, GPCR (G protein coupled receptors), myocardial infarction, cardiovascular toxicity, cardiac fibrosis

**Editorial on the Research Topic** Receptors in cardiovascular diseases: Mechanisms, diagnosis, and treatment

Cardiovascular diseases remain the leading cause of mortality worldwide. Emerging evidence has suggested a crucial role for receptors in the pathogenesis of cardiovascular diseases. Receptors mediate the communication between the extracellular and intracellular environment, transducing external signals to produce internal responses. Numerous receptors have been characterized, depending on their structure and function, including channel-linked receptors, enzyme-linked receptors, G-protein coupled receptors (GPCRs), and nuclear receptors, suggesting a great diversity in mechanisms. Aberrant receptor activation, either primary or secondary to cardiovascular diseases, may facilitate and/or exacerbate disease progression. It is therefore imperative to understand the role of the various types of receptors in cardiovascular diseases to develop novel therapies to improve clinical outcomes.

We are pleased to introduce 4 outstanding articles from 24 exceptional individuals who are affiliated with 11 different institutions. These articles highlight mechanistic insights of membrane type 1-matrix metalloproteinase (MT1-MMP) in the cleavage of low-density lipoprotein receptor (LDLR) in atherosclerosis (Wang et al.), examine the efficacy and safety of sacubitril/valsartan in acute myocardial infarction (Yang et al.), illuminate the role of GPCRs in cardiac fibrosis treatment (Zhang et al.), and suggest the potential role of TRPM7 in cardiovascular toxicity (Liu et al.). These articles are briefly summarized below.

## Identiﬁcation of amino acid residues in the Mt-loop of Mt1-MMP critical for its ability to cleave low-density lipoprotein receptor

MT1-MMP has been previously shown to cleave LDLRs, which may exacerbate the development of atherosclerosis. In this original research article (Wang et al.), examines the determinants in LDLR and MT1-MMP that were crucial for this cleavage. The authors demonstrate that the substitution of isoleucine (Ile), a nonpolar amino acid, at residue 167 of the MT-loop with alanine (Ala), threonine (Thr), or lysine (Lys) resulted in the loss of ability of MT1-MMP to cleave LDLR. In contrast, the replacement of Ile with leucine (Leu), valine (Val), methionine (Met), or phenylalanine (Phe) had no effect. Overall, the investigators provide evidence that the Ile at residue 167 is important for MT1-MMP-induced LDLR cleavage.

## Efﬁcacy and safety of sacubitril/valsartan vs. valsartan in patients with acute myocardial infarction: A meta-analysis

Angiotensin-receptor-neprilysin inhibitor (ARNI) is composed of sacubitril/valsartan, which targets the renin-angiotensin-aldosterone system (RAAS) and the natriuretic peptide system. ARNI has been shown to benefit patients with heart failure (HF). In this meta-analysis (Yang et al.), questioned whether the dual treatment might be effective in patients with acute myocardial infarction (MI). Using 9 studies, involving 1,369 patients, the authors show that patients with left ventricular ejection fraction (LVEF) treated with ARNI exhibited reduced risk of major cardiac events, heart failure, readmission and improvement of cardiac function compared to patients treated with angiotensin receptor blockers (ARBs). The study also suggested that the beneficial effects observed for ARNI might be time dependent, since more effective results were observed in patients with earlier intervention.

## Targeting GPCRs to treat cardiac ﬁbrosis

Cardiac fibrosis is a common pathological process observed in almost all cardiovascular diseases, often contributing to impairment of cardiac function and organ damage. Currently, there is no FDA-approved drugs that specifically target cardiac fibrosis. In this review (Zhang et al.), sheds light on the therapeutic potential of G-coupled protein receptors (GPCRs). The authors explored downstream pathways of GPCRs involved in fibrosis regulation by fibroblasts, cardiomyocytes, and endothelial cells. Additionally, the authors critically discussed the possible crosstalk between different GPCRs and their importance in disease progression.

## Cardiovascular toxicity of tyrosine kinase inhibitors during cancer treatment: Potential involvement of TRPM7

Inhibitors of receptors tyrosine kinase (RTK), including small molecules and monoclonal humanized antibodies have been highly effective for treating numerous human cancers. However, they are also associated with cardiovascular toxicity, including hypertension, cardiac dysfunction and heart failure. In this review (Liu et al.), provides a comprehensive discussion about the potential role of the Mg^2+^ channel transient receptor potential melastatin-subfamily member 7 (TRPM7) in cancer development and cardiovascular diseases. Moreover, the authors critically discuss the possible importance of crosstalk between TRPM7 and RTK in cardiovascular physiology and disease.

We greatly appreciate the efforts of all contributing authors towards this Frontiers Research Topic entitled *Receptors in Cardiovascular Diseases* and we look forward to more novel insights on the contribution of receptors in the pathophysiology of cardiovascular diseases.

